# Effects of Bluetooth-Enabled Desk Ellipticals on Office Work Performance: Rationale, Design, and Protocol for a Randomized Trial With Overweight and Obese Adults

**DOI:** 10.2196/16275

**Published:** 2020-01-14

**Authors:** Liza S Rovniak, Marc A Adams, Christopher N Sciamanna, Lan Kong, Nicole Sullivan, Sara Costalas, Melissa Bopp, Ashley Kuzmik

**Affiliations:** 1 Division of General Internal Medicine Departments of Medicine and Public Health Sciences Pennsylvania State University College of Medicine Hershey, PA United States; 2 College of Health Solutions Arizona State University Phoenix, AZ United States; 3 Division of Biostatistics and Bioinformatics Department of Public Health Sciences Pennsylvania State University College of Medicine Hershey, PA United States; 4 Department of Kinesiology Pennsylvania State University State College, PA United States

**Keywords:** physical activity, obesity, reinforcement, environment design, built environment, occupational health, workplace, mHealth

## Abstract

**Background:**

Workplaces that provide opportunities for physical activity without requiring extra time for activity could help counteract the obesity epidemic. Desk ellipticals can contribute to activity-supportive workplace environments; however, the feasibility of engaging employees in pedaling ellipticals during simultaneous office work has not been well evaluated.

**Objective:**

We aim to present the rationale and methods from an ongoing randomized trial with overweight and obese employees that will evaluate (1) the effects of pedaling a compact desk elliptical on work performance and (2) the influence of different incentive types and schedules on desk pedaling quantity.

**Methods:**

Overweight and obese medical center employees are being recruited in dyads for a 2 (gift card type: healthier food vs Amazon) by 3 (gift card schedule: immediate incentive contingent on individual pedaling quantity; immediate incentive partially contingent on dyads’ joint pedaling quantity; and delayed noncontingent pedaling incentive) cluster randomized within-subjects factorial trial. All participants receive a Bluetooth-enabled desk elliptical for 4 weeks and access to a mobile app that provides real-time pedaling feedback. The primary aims are to assess (1) change in employee work performance from pre- to postelliptical installation via employee and supervisor ratings and (2) effects of gift card type and schedule on quantity of objectively measured desk pedaling completed.

**Results:**

Data collection is ongoing. We expect to complete main outcome analyses in 2020.

**Conclusions:**

This trial represents one of the earliest attempts to assess the effects of desk pedaling and pedaling-incentive types in real-world offices. It could help bridge the research-to-practice gap by providing evidence on whether desk pedaling can be sustained without compromising work performance.

**International Registered Report Identifier (IRRID):**

DERR1-10.2196/16275

## Introduction

### Background

Most working-aged US adults spend more than half of each day in sedentary behavior [1,2] and at least 60% do not meet recommended physical activity guidelines [3,4], which increases the risk for obesity and chronic disease [4-6]. Adults in the workforce identify lack of time as one of the most significant barriers to regular physical activity [7,8]. Fewer than 1 in 3 employed adults typically participate in workplace physical activity programs [9,10].

Desk ellipticals, which enable people to expend about 85 to 90 extra kilocalories per hour over sedentary sitting [11,12], can address the time barrier to physical activity in 2 unique ways. First, desk ellipticals reduce the opportunity cost of physical activity, as employees can pedal *while* working and are not required to commit extra time to complete physical activity [13]. Second, unlike typical workplace physical activity interventions which are conducted outside working hours [14], desk ellipticals can be placed in employees’ immediate environment—near computers, telephones, and coworkers [11]. This feature is important because lack of time for physical activity often reflects a low density of immediate cues (prompts) and reinforcers (eg, economic, social, physical, or emotional benefits) for physical activity, coupled with a higher density of such cues and reinforcers for competing work and social demands [15,16]. Placing desk ellipticals in employees’ workspaces, where they can generate immediate cues and reinforcement for activity (eg, visual prompts to pedal, coworker modeling of pedaling, or praise for pedaling), is consistent with ecological models and research supporting the importance of proximal environmental influences on physical activity and sedentary behavior [17,18].

### Desk Pedaling and Work Performance

To disseminate desk ellipticals or similar devices on a large scale, employers will require evidence that pedaling such devices does not compromise work performance [19,20]. Most previous studies that assessed simultaneous work performance during desk pedaling were conducted in lab-based settings [21-26]. These lab-based studies supported the feasibility of completing specific work tasks during simultaneous desk pedaling, within controlled environments that maximized internal validity [27]. However, there has been limited research on work performance and simultaneous desk pedaling in field settings where employees perform their actual jobs. Conducting work performance evaluations in field settings is important for the external validity of study findings, as these settings may include unique social and built environment constraints or facilitators (eg, coworker criticism or praise, and office layout variations) that are difficult to engineer in a lab [27,28].

Among several studies that investigated the use of compact elliptical or pedaling devices in real-world offices, employee-administered surveys suggested that it was feasible to work productively while engaged in simultaneous pedaling [13,29,30]. However, these studies lacked input from supervisors about the effects of pedaling devices on employee performance—which is needed to ensure more widespread acceptance and dissemination of these devices. These studies also lacked assessment of employees’ ability to perform specific common office work tasks (eg, emails and phone calls) while pedaling. Therefore, a more comprehensive assessment of work performance during office-based pedaling is warranted.

### Incentives and Desk Pedaling Quantity

Previous office-based desk pedaling studies demonstrated small declines in employees’ pedaling activity over time [13,29,31,32], suggesting a need for greater reinforcement of pedaling activity. Both primary food-based and generalized monetary reinforcers can help to sustain physical activity [33-39], but these types of incentives have not been explored in desk pedaling trials, or directly compared for their effects on sustaining physical activity or other health behaviors. Providing healthier food-based incentives may offer advantages over traditional monetary incentives or cash-equivalent gift cards, including the potential to address the obesity epidemic, increase cost-effectiveness via improved health, and build cultural norms for healthful eating [33]. In contrast with money or cash-equivalent gift cards which are obtained at a fixed price point, there may also be opportunities to secure volume discounts for food items to attain cost savings for population-wide interventions. Understanding the differential effects of healthier food versus monetary or cash-equivalent incentives could inform the design of popular workplace incentive plans for healthy lifestyle change [40].

In addition to the differential effects of incentive type, incentive delivery schedules may impact desk pedaling quantity. Food or monetary reinforcers delivered on an immediate reinforcement schedule close-in-time to achieving physical activity and other behavioral goals have been more effective in increasing physical activity or motivation than delayed reinforcers [34,41,42]. Some evidence also suggests increased likelihood of achieving behavioral health goals when the receipt of reinforcers is partially contingent on 2 or more people achieving a goal, rather than solely dependent on individual goal achievement [43,44]. The effects of varying these reinforcement schedules on desk pedaling quantity have not yet been investigated.

### Purpose and Hypotheses

In sum, we aim to present the rationale and methods for a randomized trial among overweight and obese employees that will assess the effects of (1) desk pedaling on work performance using comprehensive employee- and supervisor-rated work performance measures and (2) healthier food versus monetary incentives with varied reinforcement schedules on pedaling quantity. We hypothesize that a 4-week desk pedaling intervention period, as compared with a 4-week preintervention period of standard office sitting, will not yield meaningful differences in employees’ work performance. We also hypothesize that healthier food and monetary incentives delivered (1) on an immediate rather than a delayed schedule, and (2) partially contingent on dyadic- rather than solely individual-goal achievements, will yield greater pedaling quantity. Achieving these aims could help bridge the research-to-practice gap in translating desk ellipticals from laboratory settings to real-world office environments.

## Methods

### Design

We will use a 2 (gift card type: healthier food vs Amazon) by 3 (gift card schedule: immediate incentive contingent on individual pedaling quantity, immediate incentive partially contingent on dyads’ joint pedaling quantity, and delayed noncontingent pedaling incentive) cluster randomized within-subjects factorial design. Employee dyads (n=60 or 30 two-person clusters) will be recruited on a rolling basis to join the program together and will be randomly assigned in clusters to 1 of the 6 study groups using computer-generated permuted block randomization (block size of 6 with equal allocation, determined by a statistician).

The intervention phase will last 4 weeks, with within-subject assessments (via pre- and postintervention questionnaires) used to capture changes in work performance and reported nonpedaling physical activity between a 4-week preintervention period with standard office sitting and a 4-week intervention period with desk pedaling. The primary outcomes are (1) changes in employee- and supervisor-reported work performance between the 4-week preintervention period and the 4-week intervention period and (2) objectively measured pedaling quantity during the 4-week intervention period based on data from the Bluetooth-enabled desk ellipticals. The secondary outcomes include employee-reported changes in nonpedaling physical activity, cost-effectiveness of incentive conditions (measured by the total dollar value of gift cards distributed and redeemed, and by work productivity and body weight changes), participant satisfaction, and built and social environment influences on employees’ work performance and pedaling quantity.

The study was approved by the Pennsylvania State University College of Medicine Institutional Review Board, and the National Institutes of Health peer-review statements are included in Multimedia Appendices 1 and 2.

### Inclusion and Exclusion Criteria

Inclusion criteria for participation are shown in Textbox 1. Exclusion criteria for participation are shown in Textbox 2.

**Inclusion criteria.**

- Overweight or obese (body mass index between 25 and 55 kg/m^2^)
- Employed full time at Penn State Hershey Medical Center and physically present in the office a minimum of 35 hours per week to ensure it is feasible to complete daily pedaling
- Work between 6 am and 6 pm, Monday through Friday, as this is the time frame during which study staff are available to oversee the program
- Desk-based office job involving sedentary work for ≥5 hours per day, 5 days per week
- Use a nonshared desk so the pedaling measured can be attributed to the study participant
- Aged between 18 and 70 years
- Able to read and speak English
- Own an Android or iPhone smartphone with internet or Wi-Fi access and willing to install the free Cubii cycling app (Fitness Cubed Inc) needed for all study conditions on their smartphone
- Work in a campus building that has at least one onsite Hershey-operated food vendor. On the basis of research showing the importance of resource proximity for resource use [45,46], the food-based rewards are more likely to be used if they are easily accessible
- Able to obtain their supervisor’s approval to participate
- Able to identify a coworker to do the program together with, who also meets all eligibility criteria after separately completing the screening form, based on evidence that social environment support can promote physical activity [45,47,48]

**Exclusion criteria.**

- Currently pregnant
- Health or personal condition (eg, planned surgery) that could prevent program completion
- Physical Activity Readiness Questionnaire [49] response indicating that participants (1) have been advised that they have a heart condition and should only do physical activity recommended by their doctor or (2) have chest pain during physical activity
- Planned travel or relocation that will lead participants to be unavailable for 3 or more days during the study and that cannot be accommodated by adjusting the program dates
- Already have a cycling device or treadmill workstation at their desk

### Setting and Recruitment

The study is based at the Penn State Hershey Medical Center, a major teaching and research hospital with more than 12,000 employees in Central Pennsylvania [50]. Sedentary desk jobs at the medical center are diverse and include secretarial and administrative work, grant proposal preparation, budgetary administration, radiology and scan analysis, quality control initiatives, and faculty member and other research or clinical support positions. A brief study description was distributed via electronic newsletter to all employees to publicize the study. The study description contained a link to the secure REDCap site [51] to complete a screening form to assess inclusion and exclusion criteria. The study description was additionally emailed to administrative staff who were given the option of forwarding this information to department employees. Study information was also posted on Penn State’s research recruitment website. 

Employees who remain eligible to participate after completing the screening form are asked to provide their supervisor’s contact information. Supervisors then receive a brief study summary, together with a link to a secure Web-based REDCap form to enable them to document consent for their employee(s) to participate. Following receipt of the supervisor’s permission form, an initial study meeting is scheduled at the participant’s office. At this approximately 20-min meeting, the consent form and baseline questionnaire are administered, and the research team evaluates the participant’s desk setup to determine if modifications are required to ergonomically place the elliptical under the participant’s desk (eg, accessible electrical outlet to plug in the elliptical and relocation of underdesk items).

### Intervention Procedures

#### Common Elements

**Figure 1:**
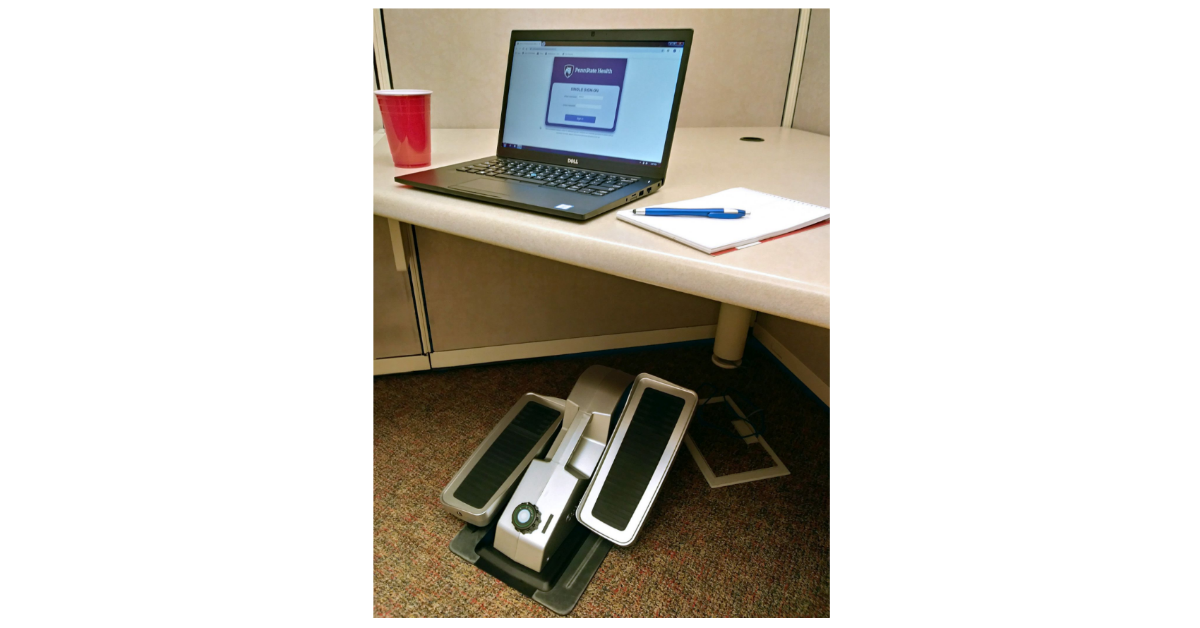
Desk elliptical setup in standard office cubicle.

Participants in all 6 conditions are provided with the Cubii Pro elliptical (Fitness Cubed Inc) at no cost (Figure 1). During an approximately 1-hour elliptical setup meeting, research staff work with each participant to set up the desk elliptical ergonomically, including placing the elliptical at a comfortable distance and angle from the participant’s chair and keyboard and equipping participants with rubber mats and chair anchors to prevent excess chair and elliptical motion while pedaling.

**Figure 2:**
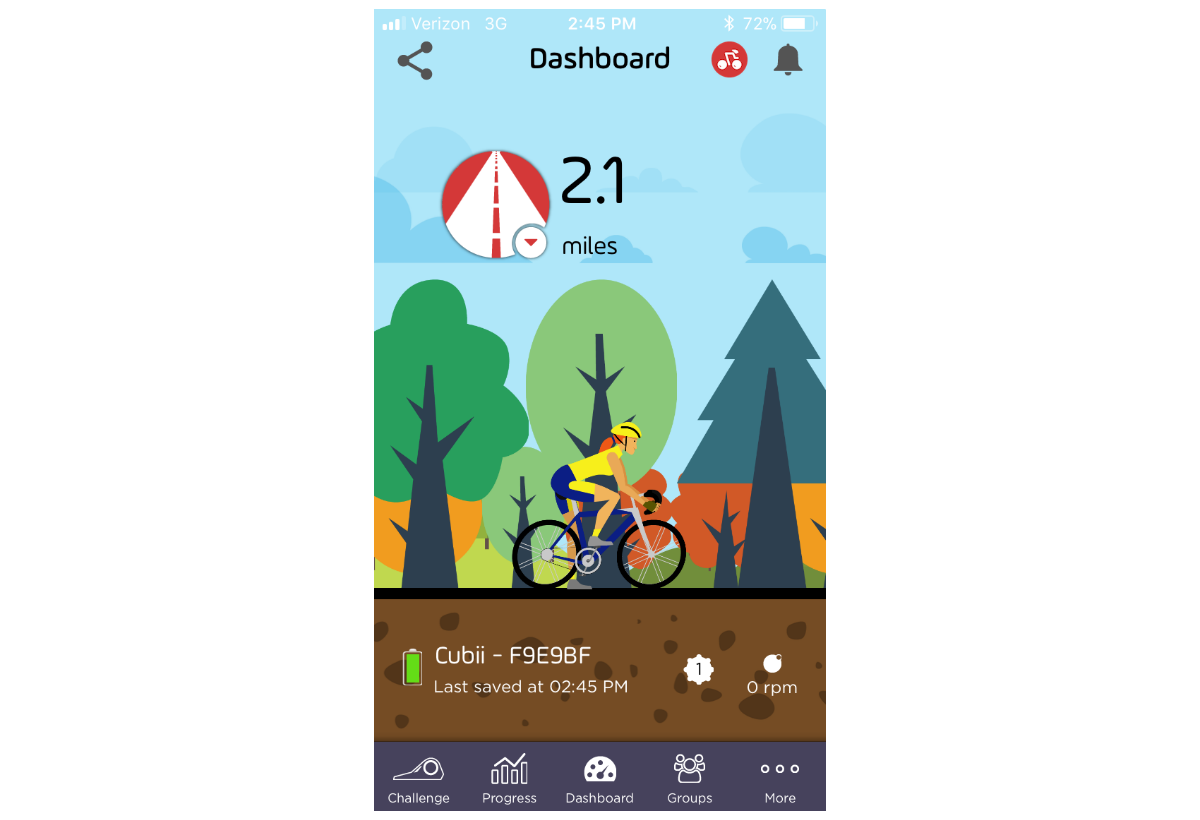
Screenshot of Cubii mobile app dashboard with real-time pedaling feedback.

During the elliptical setup meeting, research staff also install the free Cubii elliptical app on each participant’s Android or iPhone smartphone. This app provides participants with real-time automated feedback on both their own and their partner’s pedaling volume (ie, miles, strides, minutes, and calories expended), and shows a bicyclist moving in real time in sync with each participant’s pedaling (Figure 2). Research staff demonstrate key app features to participants and explain how to check the elliptical is connected to the app to prevent loss of pedaling data. Research staff also link each participant’s pedaling data to a research-administered Cubii server account and Fitbit account to enable automatic uploading of each participant’s pedaling data. Although the Cubii server data can only be obtained by the researchers on a delayed basis, participants’ pedaling data on the Fitbit site are available continuously—enabling research staff to monitor pedaling adherence in real time.

All participants are asked to attempt to pedal the elliptical at least 2 miles daily (approximately 1 hour of daily pedaling) from Monday through Friday. The goal of 2 miles, or 1 hour, of daily pedaling was determined by prior studies, which suggested that this goal could have a clinically significant impact on weight gain prevention while being feasible for overweight and unfit participants [11,13]. Participants are informed that they can choose to pedal more or less than the 2-mile goal and are advised to self-select a comfortable pedaling pace, intensity, and duration. Participants are also advised that they can pedal the desk elliptical in short bouts of a few minutes at a time or in longer bouts. Although participants are asked to attempt to pedal on all 5 workdays, they are informed that it is understandable if they occasionally need to miss a day or reduce their pedaling.

Participants receive a verbal description and handout outlining their reward type and schedule that is tailored to the study group to which they and their partner have been randomly assigned. Participants are informed in the consent document that while all participants will receive incentives, the incentive type (ie, vendor) and delivery schedule may vary depending upon which group they are randomly assigned to. To prevent treatment contamination, participants are not given procedural details regarding the incentive type and schedule that other groups receive.

#### Features Specific to Each of the Intervention Groups

**Figure 3:**
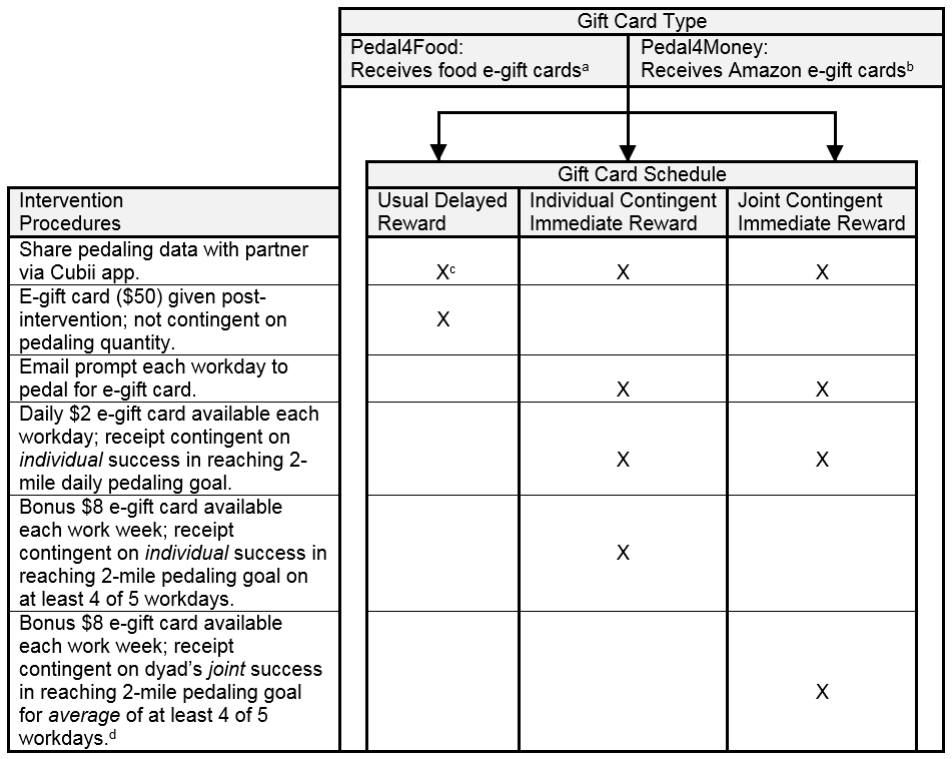
Randomized 2 (gift card type) by 3 (gift card schedule) factorial study design. a: In the Pedal4Food—Individual or Joint Contingent Immediate Reward groups, the $2 e-gift card covers a beverage (standard small coffee, tea, or bottled water), and the $8 e-gift card covers a meal (any combination of a salad, wrap, sandwich, soup, or bottled water) redeemable at the Hershey Medical Center-operated cafeterias, Au Bon Pain and Starbucks); the Usual Delayed Reward group receives $50 of combined beverage and food e-gift cards proportional in quantity to the other 2 groups; b: In the Pedal4Money—Individual or Joint Contingent Immediate Reward groups, the $2 and $8 e-gift cards can be applied toward any purchase on Amazon; the Usual Delayed Reward group receives incentives combined as a $50 Amazon e-gift card; c: The symbol “X” indicates that an intervention procedure was administered for the designated study group; d: This bonus is received if both partners meet pedaling goals on at least 4 workdays, or if one partner meets pedaling goals for 3 workdays and one meets goals for 5 workdays.

Figure 3 summarizes key details for each of the 6 intervention groups. More specific procedural details are provided below.

**Figure 4:**
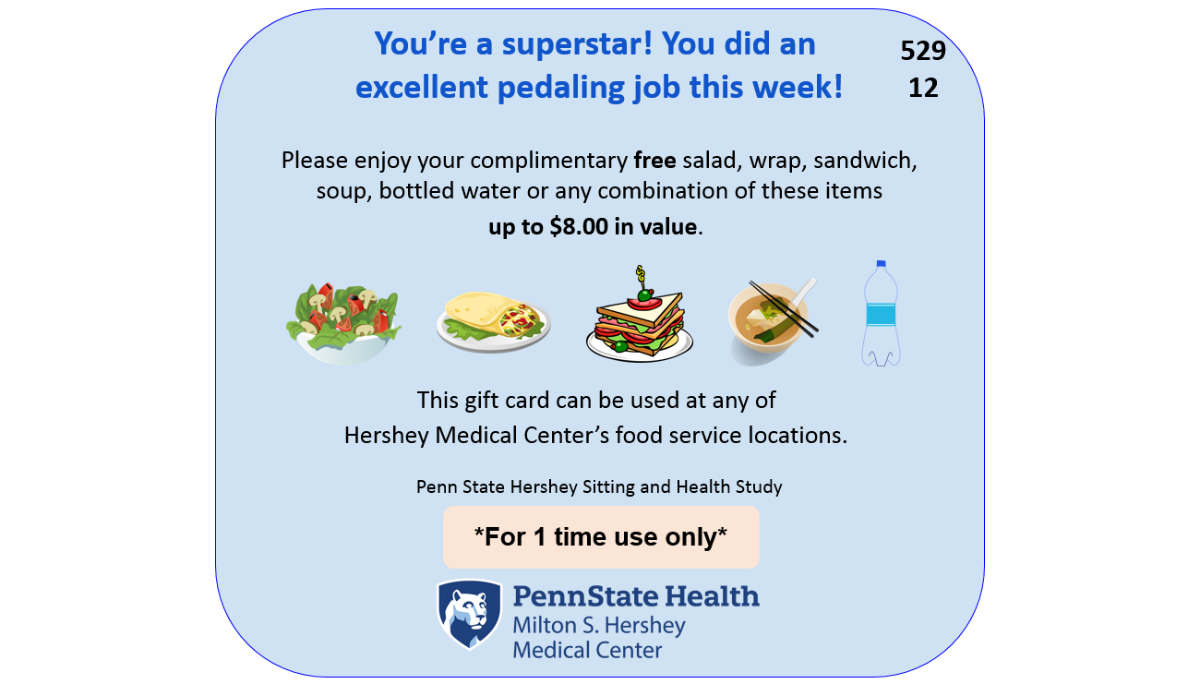
Sample e-gift card: Pedal4Food—Individual Contingent Immediate Reward condition.

Individual Contingent Immediate Reward (ICIR) and Joint Contingent Immediate Reward (JCIR) participants receive daily reminder emails to notify research staff when they reach the 2-mile pedaling goal by using the single-click Cubii app notification feature to email staff an automatically generated summary of their pedaling mileage. Each daily reminder includes a brief tip (eg, “Desk cycling can help people deal with daily life hassles and stressors!”). Research staff typically send each participant a reward email with an e-gift card (Figure 4) within 2 hours after participants submit a notification that they reached or exceeded the 2-mile daily pedaling goal.

To redeem gift cards, Pedal4Food group participants are asked to print each gift card and hand it to the Penn State-Hershey food service cashier. Participants and cashiers are instructed that the food gift cards may only be used to redeem the specific beverage or food items displayed on each gift card. All food gift cards are stapled to each participant’s food receipt and held for weekly pickup by research staff, who then manually record each card’s ID number and the items purchased. The Pedal4Money group redeems gift cards by applying their Amazon gift card codes at the Amazon website toward any purchase; redemption is tracked via Amazon by research staff.

The value of compensation over the 4-week intervention period for both the ICIR and JCIR groups ranges from US $0 (if zero pedaling is done) to US $72 (if participants meet all pedaling goals: 5 workdays×US $2=US $10, plus US $8 bonus=US $18/week×4 weeks=US $72). The maximum weekly compensation rate of US $18 was based on systematic reviews which suggested that this amount is representative of average incentive sizes and is associated with increased physical activity [38,39]. Assuming the ICIR and JCIR groups attain typical physical activity adherence rates of approximately 65% to 70% that were observed in prior trials with financial incentives [41,52-54], we expect that most ICIR and JCIR participants will earn approximately US $50—equivalent to the US $50 compensation amount provided to the Usual Delayed Reward participants.

### Measures

The study’s measures are shown in Table 1. The objective measures for pedaling volume are obtained from the Cubii company’s server and provided by the Cubii company to our research team via password-protected files. Aside from the objective Cubii measures and the gift card redemption receipts and records, all other measures are administered via secure Web-based REDCap surveys [51]. Employee participants are compensated at the end of the study US $10 in cash for completing the preprogram survey and US $15 in cash for completing the postprogram survey. Employees’ supervisors are not compensated for survey completion. Supervisors are asked to *not* share their ratings of each employee’s work performance with employees.

**Table 1:** Measures and measurement schedule.

Measures		Schedule		
		M0^a^	M1^b^	M2^c^
**Employee ratings of work performance**				
	1. The World Health Organization Health and Work Performance Questionnaire: Employees rate overall work performance in previous 4 weeks on a 10-point scale; lower employee-rated work performance associated with odds of lower performance in supervisor evaluations and records and experience sampling across multiple job types (odds ratios: 3.2-12.3, *P* values<.05) [55].	X^d^	—^e^	X
	2. Employees rate work quantity, work quality, and interaction quality in previous 4 weeks on a 5-point scale; evidence of face validity [56]; higher work quantity associated with higher physical fitness (*P*=.045), higher work quality associated with higher moderate physical activity (*P*=.002), lower interaction quality associated with greater obesity (*P*=.02) [57].	X	—	X
	3. Work performance by task type: Employees rate ability to perform common work tasks (eg, emails and phone calls) during elliptical use in previous 4 weeks, using a 5-point investigator-generated scale.	—	—	X
**Supervisor ratings of employee work performance**				
	1. The World Health Organization Health and Work Performance Questionnaire: Slightly adapted for supervisor ratings from above employee version [55].	X	—	X
	2. Supervisors rate employees’ work quantity, work quality, and interaction quality: slightly adapted from above employee version [56,57].	X	—	X
**Elliptical pedaling volume**				
	1. Objectively measured pedaling output from Cubii elliptical: total pedaling miles, strides, minutes, and calories expended.	—	X	—
	2. Percentage achieving daily 2-mile pedaling goals.	—	X	—
**Gift card distribution, redemption, and costs**				
	1. Number of gift cards distributed, by amount and type.	—	X	—
	2. Percentage of food gift cards redeemed, via food service receipts.	—	X	X
	3. Percentage of Amazon gift cards redeemed, via Amazon website.	—	X	X
**Total nonpedaling physical activity**				
	1. Stanford Leisure-Time Activity Categorical Item: Participants select 1 of 6 categories to describe their physical activity in the previous month; test-retest Spearman ρ=0.80 [58], associated with accelerometer-measured moderate-vigorous activity min/week, Spearman ρ=0.40, *P*<.001 [59].	X	—	X
	2. Global Physical Activity Questionnaire: Captures domain-specific physical activity in typical week; test-retest Spearman ρ=0.67-0.81 [60], associated with accelerometer-measured moderate-vigorous activity min/day (r=0.48; *P*<.005) [61].	X	—	X
**Participant satisfaction**				
	1. Investigator-generated program evaluation measures [62].	—	—	X
	2. Qualitative, open-ended user evaluation questions.	—	—	X
**Built and social environment**				
	1. Office Spatial Layout: Employees rate office environment features (eg, office layout and coworker proximity) on a 5-point scale; test-retest intraclass correlation coefficient=0.70-0.87, associated with occupational sitting (*P*<.05) [63].	X	—	—
	2. Employees rate reactions of coworkers, supervisor, family members, and friends to their elliptical use on a 5-point investigator-generated scale.	—	—	X
**Demographic and health characteristics**				
	1. Employee demographics, self-rated health, height, and weight.	X	—	X
	2. Supervisor demographics.	X	—	—

^a^M0=preintervention.

^b^M1=4-week intervention phase.

^c^M2=postintervention.

^d^The symbol “X” indicates that a measure was administered at this assessment point.

^e^A measure was not administered at this assessment point.

### Statistical Analysis

We will perform descriptive analyses for all measured variables. We will examine data normality and skewness, along with missing data and address any identified issues using standard procedures [27].

#### Effects of Desk Pedaling on Work Performance

For the study’s first goal, to test whether mean work performance scores during the 4-week desk pedaling intervention period are equivalent to performance during the 4-week preintervention period with standard office sitting, we will apply the equivalence test of means based on 2 one-sided *t* tests [64,65] with the significance level adjusted for multiple comparisons via the Bonferroni correction factor. We will also use the confidence interval approach for testing equivalence when regression models are considered [66].

We define equivalence based on 2 related standards: (1) the International Organization of Standardization ergonomic standard for computer keyboards indicates that average typing speeds obtained using a new keyboard must not exceed 0.75 standard deviations of average speeds for standard keyboards (in the direction of poorer performance) to be acceptable [67]; (2) in clinical research, a change of 0.50 standard deviations in health status sometimes is used as a basis for treatment modifications [68,69]. Using the approximate midpoint of these 2 standards, we define equivalence, or feasibility, for the desk elliptical as average work performance scores that do not exceed 0.60 standard deviations (in the direction of poorer performance) of average work performance scores obtained during standard office sitting.

We will also explore how work performance varies by tertiles of elliptical pedaling quantity. To evaluate changes in overall work performance, and work performance by task type, which are repeatedly measured, we will plot mean scores over time and conduct longitudinal analysis based on mixed effects models [70]. We will use random effects to account for measurement correlation within the same subject and clustering effects. The estimated time effect from mixed effects models will indicate whether performance increased, decreased, or remained stable over the preintervention and intervention periods.

For the qualitative assessment of participants’ capacity to pedal and work simultaneously, NVivo software (QSR International) will be used to organize data from the qualitative open-ended questions. A codebook will be developed to classify major themes, and data will be coded by 2 independent coders. Coding discrepancies will be discussed and resolved, and interrater reliability will be calculated.

#### Effects of Incentive Condition on Desk Pedaling Quantity

For the study’s second goal, to assess the effects of the 6 incentive conditions on pedaling quantity (miles and minutes) completed over 4 weeks, we will model elliptical miles and minutes per day as continuous outcomes using linear mixed effects models with repeated observations of each outcome variable (level 1) treated as nested within (n=60) individual participants (level 2), while accounting for clustering effects within each dyad (level 3). We will add to our models effect coded vectors for gift card type (food vs Amazon) and gift card schedule (Usual Delayed Reward vs ICIR vs JCIR) to test for main effects; along with gift card type×gift card schedule interaction terms to test for simple effects across cells.

Covariates will be included in all analyses to adjust for participants’ demographic and health characteristics (eg, age, gender, race and ethnicity, education, body mass index, and nonpedaling physical activity). Finally, we will use multivariable mixed effects models to explore the association of employees’ demographic and health characteristics, social and built-office environment factors, and supervisor characteristics with employees’ work performance and pedaling quantity. All mixed effects models will follow an intention-to-treat principle, using all available data.

Other secondary analyses will depend on the specific research question and the most appropriate statistical or qualitative methods for the design.

#### Power and Sample Size

We assume that an equivalence margin standardized by the standard deviation is 0.60 for the primary outcome, based on clinical and industrial engineering standards [67-69]. We also assume an intracluster correlation of 0.05 and a significance level of 0.05. Therefore, a sample size of 50 (25 dyads) gives 80% power to detect equivalence when assessing the mean delta change in total work performance scores from the preintervention period to the intervention period. Anticipating 10% to 20% attrition, we expect to recruit up to 60 participants. The study was not powered to detect differences in pedaling volume by the 6 incentive conditions because of resource constraints and because a key exploratory goal was to assess feasibility and preliminary effects of different incentive strategies.

## Results

Data collection will be completed by December 2019. We expect to complete main outcome analyses in 2020.

## Discussion

### Principal Considerations

Since 1960, increased computer automation in the workplace has led average work-related energy expenditure to drop by more than 100 calories per day [71]. This progressive decline in working adults’ daily energy expenditure has contributed to rising obesity rates, with 40% of US adults currently obese [71-73]. Adults in small-to-medium size metropolitan statistical areas, such as Central Pennsylvania, are at even greater risk for obesity than adults in more urbanized regions [74], indicating a need for wider environmental support to promote employee health. Desk ellipticals, which are compact, relatively low-cost, and scalable across diverse workplaces, may contribute to creating healthier workplace environments—consistent with the goals of the National Institutes of Health Total Worker Health Initiative [75]. This study aimed to respond to the need to create healthier workplaces to prevent or reduce overweight and obesity by conducting a randomized trial to assess the feasibility of engaging employees in pedaling desk ellipticals while simultaneously completing productive office work. The knowledge gained from this study may help guide efforts to create environments and policies that promote *active* office work as a standard feature of occupational health practice.

### Strengths and Limitations

Strengths of this study include its use of supervisor ratings of employee work performance and objective measures of pedaling quantity and gift card redemption. Limitations of this study include its short duration and use of a small convenience sample of employees at a single worksite. An intervention period of 4 weeks was selected given resource constraints and to maximize the likelihood of supervisors agreeing to permit employees to participate in this novel intervention. Future studies should use a longer intervention duration to obtain more complete information about the effects of desk pedaling on work performance and the effects of different feedback and incentive strategies on employees’ pedaling volume. Furthermore, our goal was to obtain estimates of the most effective incentive strategies before automation; however, future trials could increase efficiency by automating incentive delivery.

### Comparison With Prior Work

Previous research indicates that employees can pedal desk pedaling devices without detrimental effects on objectively measured nonpedaling physical activity [30,76] and employee-rated work performance [13,29,30], and that there is interest in using these devices among adults with multiple health risk factors [77]. Our study adds to this early literature by including measures important for guiding wider dissemination of desk pedaling devices, including supervisor ratings of employee work performance, the differential effects of different incentive strategies on pedaling volume, social and built-office environment influences on desk pedaling, and qualitative assessment of user-encountered issues. Our study also allows enrollment of participants with greater health risks than most previous similar studies, which can inform the real-world generalizability of desk pedaling.

### Conclusions

Demonstrating that people can simultaneously pedal compact devices and work productively, and that they are willing to sustain this pedaling, will set the stage for future trials to (1) track longer term effects of desk pedaling on health outcomes and work performance in diverse populations and (2) evaluate effects of automated real-time feedback and incentive systems to sustain desk pedaling. Ultimately, these initiatives will grow the evidence base needed to build workplaces that support active lifestyles as a normative occupational practice.

## Appendix

Multimedia Appendix 1 First summary statement from National Institutes of Health peer review. Our proposal was funded to conduct two studies: a lab study and a field study. This manuscript describes the protocol for the field study.

Multimedia Appendix 2 Second summary statement from National Institutes of Health peer review.

## Abbreviations

ICIR: Individual Contingent Immediate Reward
JCIR: Joint Contingent Immediate Reward
