# Supplementary material for: Effects of Bluetooth-Enabled Desk Ellipticals on Office Work Performance: Rationale, Design, and Protocol for a Randomized Trial With Overweight and Obese Adults
Source: JMIR Res Protoc. 2020 Jan 14;9(1):e16275. doi: 10.2196/16275 (PMC6996735; doi:10.2196/16275)
Supplement: Multimedia Appendix 1 [file resprot_v9i1e16275_app1.pdf]

SUMMARY STATEMENT
(Privileged Communication)

PROGRAM CONTACT:
Charlotte Pratt
(301) 435-0382
prattc@nhlbi.nih.gov

Release Date: 10/24/2012

Application Number: 1 R21 HL118453-01

Principal Investigator

ROVNIAK, LIZA PHD

Applicant Organization: PENNSYLVANIA STATE UNIV HERSHEY MED CTR

Review Group: PRDP
Psychosocial Risk and Disease Prevention Study Section

Meeting Date: 10/02/2012
Council: JAN 2013
Requested Start: 04/01/2013

RFA/PA: PA12-179
PCC: HHCG N
Dual PCC: NLM DUAL
Dual IC(s): DK

Project Title: Desk-Compatible Elliptical Device: Feasibility Evaluation

SRG Action: Impact Score: 28 Percentile: 23 +
Next Steps: Visit http://grants.nih.gov/grants/next_steps.htm
Human Subjects: 44-Human subjects involved - SRG concerns
Animal Subjects: 10-No live vertebrate animals involved for competing appl.
Gender: 1A-Both genders, scientifically acceptable
Minority: 1A-Minorities and non-minorities, scientifically acceptable
Children: 1A-Both Children and Adults, scientifically acceptable
Clinical Research - not NIH-defined Phase III Trial

Project Year	Direct Costs Requested	Estimated Total Cost
1	150,000	229,499
2	125,000	191,250
TOTAL	275,000	420,749

ADMINISTRATIVE BUDGET NOTE: The budget shown is the requested budget and has not been adjusted to reflect any recommendations made by reviewers. If an award is planned, the costs will be calculated by Institute grants management staff based on the recommendations outlined below in the COMMITTEE BUDGET RECOMMENDATIONS section.

PROTECTION OF HUMAN SUBJECTS UNACCEPTABLE

RESUME AND SUMMARY OF DISCUSSION: This application requests support to test the feasibility of a desk compatible elliptical device to promote exercise at the worksite. It offers high impact to address the public health challenge of sedentary lifestyles. Reviewers were very enthusiastic about this work, with a minority opinion voicing concerns about the approach. Reviewers noted many strengths: the outstanding significance of the research question; the exceptional investigators and research environment; the outstandingly novel approach to increasing worksite exercise inherent in an elliptical device installed at a worker's desk; the ready and inexpensive dissemination this work could have to impact occupational health; and a compelling lab and field trial, cross over design, excellent recruitment plan and assessment of work productivity. Some weaknesses discussed: missed opportunity to conduct qualitative data collection. A minority opinion noted concerns that the feasibility metrics were missing and that the field study length of five days was insufficient to be useful. Other reviewers disagreed. Overall, reviewers concluded this was an outstanding study that could greatly inform future research, with a few weaknesses that could readily be addressed.

DESCRIPTION (provided by applicant): The proposed research will investigate the feasibility of accomplishing simultaneous caloric expenditure and productive office work using a height-adapted elliptical device designed to be pedaled at a standard desk. Strategies for increasing energy expenditure without requiring extra time investment are urgently needed, as most US adults in the workforce report "lack of time" for exercise, and spend over half of their waking hours in sedentary behavior. These low levels of energy expenditure contribute to an average weight gain of 1 pound per year among US adults-which raises risk for cardiovascular disease, metabolic dysfunction, diabetes, and cancer. Pedaling a low-cost elliptical device while simultaneously completing office work could help reverse this trend toward weight gain-without requiring extra time investment to exercise. However, there is a lack of research on the feasibility of accomplishing productive office work while using elliptical/pedaling devices. Evaluating the feasibility of simultaneous pedaling and productive office work is important for determining whether elliptical/pedaling devices should be more widely disseminated across diverse sedentary office settings. Therefore, the primary specific aims of this research are to: (1) assess the feasibility of completing simulated office work activities in a lab-based setting while pedaling the elliptical at different intensity levels among sedentary adults varying on age, gender, and body mass index (Study 1, n = 132); and (2) assess the feasibility of completing sedentary desk work in a field-based (office) setting while pedaling the elliptical at a self-selected intensity level, as well as social environment influences on elliptical use (Study 2, n = 30). The proposed research builds upon established ecological models demonstrating the importance of proximal environmental influences on physical activity and sedentary behavior. The combination of lab- and field-based research will contribute to the internal and external validity of study findings, and help guide recommendations for integrating elliptical/pedaling devices into sedentary office settings. Even if used only part of the working day, elliptical devices could substantially increase daily caloric expenditure and contribute to reduced risk of obesity and chronic health conditions associated with sedentary lifestyles.

PUBLIC HEALTH RELEVANCE: The proposed research will explore the feasibility of accomplishing simultaneous energy expenditure and productive office work while pedaling a desk-compatible elliptical device. Unlike other existing solutions for promoting simultaneous office work and energy expenditure, the desk-compatible elliptical device is low cost, space-efficient, and can be used while seated in a standard office chair at a standard-height work desk. Widespread use of low-cost desk-compatible ellipticals, or similar devices, could help prevent further growth of the obesity epidemic and reduce risk of cardiovascular disease.

CRITIQUE 1:

Significance: 2
Investigator(s): 2
Innovation: 3
Approach: 6
Environment: 1

Overall Impact: The application presents a fairly innovative approach to addressing a very important public health problem. Dr. Rovniak and colleagues are well qualified to complete the proposed study. The research environment at Penn State is excellent. Strengths of the approach include the preliminary research and the analytic design of the lab study. There were a few concerns including operationalizing feasibility, reconsidering the definition of sedentary, and the usefulness of the field study.

1. Significance:

Strengths

- The proposed study is significant as it will examine the feasibility of installing in the work environment a height-adapted elliptical device designed to be pedaled at a standard work desk. The objective is to increase energy expenditure with the ultimate goal of preventing and reducing obesity and promoting overall health.

Weaknesses

- None noted.

2. Investigator(s):

Strengths

- Dr. Rovniak and colleagues, with expertise in behavioral science and physical activity, bioengineering and ergonomics, and biostatistics, have the training and experience to complete the proposed study.

Weaknesses

- None noted.

3. Innovation:

Strengths

- The desk-compatible elliptical device is fairly innovative because it overcomes some of the limitations of other devices and programs that are designed to increase physical activity in the work environment.

Weaknesses

- None noted.

4. Approach:

Strengths

- The preliminary studies strengthen the application.
- The analytic design (four-period crossover design- 4 intensity levels) for the lab study is well thought out.
- Consideration of the issues with participant recruitment is a strength of the approach.

- The approach includes a thoughtful selection of measures (notwithstanding the comment below about assessing feasibility).
- A strength of the application is that there are clear plans for how the proposed study will inform future research.

Weaknesses

- The research project is initially presented as two feasibility studies. However, the definition of feasibility in the context of either study is not stated. Also, it is not indicated which measures will be used to determine feasibility. Finally, it is not clear how the statistical analyses (for both studies but especially for the field setting study) will inform the decision regarding if the desk-compatible elliptical device is feasible or not feasible for the worksite. The approach is more similar to an outcome study rather than a feasibility study.
- The participant inclusion criteria regarding being sedentary could be reconsidered. Individuals who are physically active at some level but less than 150 minutes per week of moderate-intensity physical activity or less than 60 minutes per week of vigorous-intensity activity are not sedentary. A wide range of physical activity behavior falls under the study's definition of sedentary; and the wide definition could confound the study's results.
- The goal is to ensure that the study population is representative of normal weight and obese individuals by recruiting both types of participants. However, these individuals may be fundamentally different in ways that may limit the power of the study to detect significant differences.
- It's stated that the lab study is powered for the primary outcome (computer typing speed). Is it also powered for the secondary outcomes (cognitive processing performance, preferred pedaling intensity, and future interest in elliptical use)?
- The field study has a very short duration (5 days) that may yield little useful information due to, if nothing else, the fact that the novelty of the device may persist for the short time period. It appears the intervention period could be extended to as long as 3 months of potential pedaling by office workers. That would present a much more rigorous and meaningful evaluation of the elliptical device.

5. Environment:

Strengths

- The environment at Penn State is more than adequate to complete the proposed study.
- There are letters of support including a letter from Dr. Freivalds who is a study co-investigator and the Director of the lab where the first study will be conducted.

Weaknesses

- None noted.

Protections for Human Subjects:

Acceptable Risks and/or Adequate Protections

- The risks are minimal and adequate protections are in place.

Data and Safety Monitoring Plan (Applicable for Clinical Trials Only):

Unacceptable

  - There is no DSMP.

Inclusion of Women, Minorities and Children:

G1A - Both Genders, Acceptable

M1A - Minority and Non-minority, Acceptable

C1A - Children and Adults, Acceptable

- Equal numbers of females and males will be recruited. A significant number of minorities will participate. Participants as young as 20 years of age will be included into the study.

Vertebrate Animals:

Not Applicable (No Vertebrate Animals)

Biohazards:

Not Applicable (No Biohazards)

Budget and Period of Support:

Recommend as Requested

CRITIQUE 2:

Significance: 1
Investigator(s): 1
Innovation: 1
Approach: 1
Environment: 1

Overall Impact: The investigators aim to test the feasibility of accomplishing simultaneously caloric expenditure and productive office work using a height adapted elliptical device designed to be pedaled at a standard desk. The investigators aim to assess the feasibility in a lab based setting while pedaling at different intensity levels among a study population of sedentary adults with variation in age, gender, and BMI. In addition, the investigators have planned for a second study where the feasibility of completing desk work while pedaling the elliptical at self-selected intensity levels will be measured in office environments. The application is well written and lead by a strong investigative team. The studies are well designed and supported by previous research. If successful, the public health significance of this easy to use and economical intervention will be high in increasing physical activity within sedentary office environments.

1. Significance:

Strengths

- The investigators are addressing the current trend where work related caloric expenditure has decreased over the past several decades and many people in the work force are in sedentary office jobs.
- The aim to develop desk compatible elliptical devices makes increasing daily exercise easy and affordable.

- The desk compatible elliptical device is consistent with recommendations from both the CDC and American Heart Association.

Weaknesses

- None noted.

2. Investigator(s):

Strengths

- The study is led by a strong investigative team.

Weaknesses

- None noted.

3. Innovation:

Strengths

- The device is innovative in that it is an improvement on existing workplace exercise devices in that it: (a) has the potential to be used without leaving one's desk, increasing the convenience of use; and (b) is inexpensive and therefore feasible for employers to invest in if effective.
- It is innovative to measure the effect of the device use on work productivity as that would be a concern if the device were to be used with any regularity.

Weaknesses

- None noted.

4. Approach:

Strengths

- The design to conduct both a lab study where intensity levels and work performance measures can be controlled in addition to an observational study where the effect of self- selected intensity on work performance is strength.
- The work performance measures seem acceptable.
- The measurement of future interest in the use of the elliptical is strength in measuring the feasibility of this type of device use in work environments.
- The use of an Actigraph for measurement of the elliptical use is strength in the second aim.
- The measurement of the social influences within real work environments is also strength.
- The analytical plans for both aims are well described.

Weaknesses

- None noted.

5. Environment:

Strengths

- The environment at Pennsylvania State University is well suited for the research.

Weaknesses

- None noted.

Protections for Human Subjects:

Acceptable Risks and/or Adequate Protections

Data and Safety Monitoring Plan (Applicable for Clinical Trials Only):

- A Data Safety Monitoring Plan should be provided.

Inclusion of Women, Minorities and Children:

G1A - Both Genders, Acceptable

M1A - Minority and Non-minority, Acceptable

C3A - No Children Included, Acceptable

Vertebrate Animals:

Not Applicable (No Vertebrate Animals)

Biohazards:

Not Applicable (No Biohazards)

Budget and Period of Support:

Recommend as Requested

CRITIQUE 3:

Significance: 3
Investigator(s): 2
Innovation: 1
Approach: 3
Environment: 1

Overall Impact: This application will investigate the feasibility of using an under-desk elliptical device to provide physical activity during work. The study includes both a tightly controlled laboratory trial testing the impact of pedaling on both working speed (typing) and cognitive tasks, and a second field trial examining the social and environmental impact of elliptical exercise in a real world work environment. The study is quite well designed and there is good justification for conducting it. Assessments and study procedures are well described and analytic plans are generally good. Absence of qualitative work (e.g., focus groups and/or interviews) lessens enthusiasm somewhat.

1. Significance:

Strengths

- Americans are under-active and increasingly involved in sedentary work that creates a barrier to physical activity (PA).

- Existing PA devices at work are either ineffective (ball chairs), too expensive and/or too cumbersome to be practical. The device tested overcomes these barriers.
- Tests the impact of using a PA device on real-world work productivity

Weaknesses

- None noted.

2. Investigator(s):

Strengths

- The Principal Investigator has a PhD in clinical psychology and MPH degree. She has an excellent background in physical activity research and relevant publications, and has been Principal Investigator on two NIH grants.
- Co-investigators have degrees and experience in bioengineering and biostatistics.
- This team has conducted preliminary work that is relevant to this specific application

Weaknesses

- No expertise in qualitative data collection is present on the team.

3. Innovation:

Strengths

- Several studies have examined work-place devices for promoting PA, but none have worked with this particular elliptical modification. None have used both laboratory and field comparisons.

Weaknesses

- None noted.

4. Approach:

Strengths

- The rationale for the study is well documented
- Aims and objectives are clearly described and reasonable.
- Use of a randomized cross-over design using Balance Latin Squares is an excellent choice
- Outcome measures for the lab study are clear, reasonable and well defined.
- Use of both typing speed and a variety of cognitive tasks greatly enriches the potential findings from this study.

Weaknesses

- It is unclear what the investigators mean by “age categories” (bottom page 33).
- Pedal practice sessions are not well described.
- Lack of focus groups and/or interviews greatly limits the quality and depth of any qualitative data they might collect. Such data would seem especially important in formative research such as this.

- It’s unclear why the number of co-workers that the participants talked to about this device is of interest more likely an artifact of having to explain the equipment under one’s desk, rather than an indicator of enthusiasm.

5. Environment:

Strengths

- Resources and facilities at Penn state are more than ample to conduct this research.

Weaknesses

- None noted.

Protections for Human Subjects:

Acceptable Risks and/or Adequate Protections

- Risks are minimal in this study

Data and Safety Monitoring Plan (Applicable for Clinical Trials Only):

Unacceptable

- None provided

Inclusion of Women, Minorities and Children:

G1A - Both Genders, Acceptable

M1U - Minority and Non-minority, Unacceptable

C1A - Children and Adults, Acceptable

- Both genders will be equally represented in this study. Plans to recruit and ensure adequate representation of minorities are inadequate, and seem to be cut & pasted from a different application. They state that they will target employers with adequate minority representation in the workforce, however the employer has already been identified, and community recruitment is not applicable for this study. Children age 20 will be included along with adults age 21 and older.

Vertebrate Animals:

Not Applicable (No Vertebrate Animals)

Biohazards:

Not Applicable (No Biohazards)

Budget and Period of Support:

Recommend as Requested

Additional Comments to Applicant (Optional):

- The project timeline includes time for grant writing, which is not usually allowable

THE FOLLOWING RESUME SECTIONS WERE PREPARED BY THE SCIENTIFIC REVIEW OFFICER TO SUMMARIZE THE OUTCOME OF DISCUSSIONS OF THE REVIEW COMMITTEE ON THE FOLLOWING ISSUES:

PROTECTION OF HUMAN SUBJECTS (Resume): UNACCEPTABLE. The investigators are requested to provide a fully detailed Data Safety and Monitoring Plan to assure full protection from all risks for all study participants.

INCLUSION OF WOMEN PLAN (Resume): ACCEPTABLE. Both females and males will be recruited equally for this study.

INCLUSION OF MINORITIES PLAN (Resume): ACCEPTABLE. The target enrollment estimates inclusion of all race and ethnic 7 percent Hispanic, 7 percent Black, 3 percent American Indian and 2 percent Native Hawaiian participants.

INCLUSION OF CHILDREN PLAN (Resume): ACCEPTABLE. Children and adults aged 20 and older will be recruited for this study.

COMMITTEE BUDGET RECOMMENDATIONS: The budget was recommended as requested.

+ Derived from the range of percentile values calculated for the study section that reviewed this application.

NIH has modified its policy regarding the receipt of resubmissions (amended applications). See Guide Notice NOT-OD-10-080 at http://grants.nih.gov/grants/guide/notice-files/NOT-OD-10-080.html.
The impact/priority score is calculated after discussion of an application by averaging the overall scores (1-9) given by all voting reviewers on the committee and multiplying by 10. The criterion scores are submitted prior to the meeting by the individual reviewers assigned to an application, and are not discussed specifically at the review meeting or calculated into the overall impact score. For details on the review process, see http://grants.nih.gov/grants/peer_review_process.htm#scoring.

MEETING ROSTER

Psychosocial Risk and Disease Prevention Study Section
Risk, Prevention and Health Behavior Integrated Review Group
CENTER FOR SCIENTIFIC REVIEW
PRDP
October 02, 2012 - October 03, 2012

CHAIRPERSON

KEEFE, FRANCIS J, PHD
PROFESSOR
DEPARTMENT OF PSYCHIATRY
AND BEHAVIORAL SCIENCES
DUKE UNIVERSITY MEDICAL CENTER
DURHAM, NC 27710

MEMBERS

ABRANTES, ANA M, PHD
ASSISTANT PROFESSOR
DEPARTMENT OF PSYCHIATRY AND
HUMAN BEHAVIOR, ALPERT MEDICAL
SCHOOL, BUTLER HOSPITAL
BROWN UNIVERSITY
PROVIDENCE, RI 02906

AYALA, GUADALUPE X, PHD
PROFESSOR
INSTITUTE FOR BEHAVIORAL AND
COMMUNITY HEALTH
SAN DIEGO STATE UNIVERSITY
SAN DIEGO, CA 92123

BAEZCONDE-GARBANATI, LOURDES ALBERTINA, PHD
ASSOCIATE PROFESSOR
DEPARTMENT OF PREVENTIVE MEDICINE
AND SOCIOLOGY, KECK SCHOOL OF MEDICINE
NORRIS COMPREHENSIVE CANCER CENTER
UNIVERSITY OF SOUTHERN CALIFORNIA
LOS ANGELES, CA 90045

BARTLETT, SUSAN J, PHD
ASSOCIATE PROFESSOR
DIVISION OF CLINICAL EPIDEMIOLOGY
ROYAL VICTORIA HOSPITAL
MCGILL UNIVERSITY
MONTREAL, QC H3A 1A1
CANADA

BASEN-ENGQUIST, KAREN M, PHD *
PROFESSOR
DEPARTMENT OF BEHAVIORAL SCIENCE
M.D. ANDERSON CANCER CENTER
UNIVERSITY OF TEXAS
HOUSTON, TX 77230

BASKIN, MONICA L, PHD
ASSOCIATE PROFESSOR
DIVISION OF PREVENTIVE MEDICINE
UNIVERSITY OF ALABAMA AT BIRMINGHAM
BIRMINGHAM, AL 35294

BOCK, BETH C, PHD
PROFESSOR
CENTER FOR BEHAVIORAL AND
PREVENTIVE MEDICINE
MIRIAM HOSPITAL
BROWN MEDICAL SCHOOL
PROVIDENCE, RI 02903

BOUTELLE, KERRI N, PHD
ASSOCIATE PROFESSOR
DEPARTMENT OF PEDIATRICS
AND PSYCHIATRY
UNIVERSITY OF CALIFORNIA, SAN DIEGO
LA JOLLA, CA 92037

BRANSCUM, PAUL WESLEY, PHD *
ASSISTANT PROFESSOR
DEPARTMENT OF HEALTH
AND EXERCISE SCIENCE
THE UNIVERSITY OF OKLAHOMA
NORMAN, OK 73019

COUPS, ELLIOT J, PHD
ASSOCIATE PROFESSOR
ROBERT WOOD JOHNSON
MEDICAL SCHOOL
THE CANCER INSTITUTE OF NEW JERSEY
NEW BRUNSWICK, NJ 08901

DAVISON, KIRSTEN , PHD
ASSOCIATE PROFESSOR
DEPARTMENT OF NUTRITION
HARVARD SCHOOL OF PUBLIC HEALTH
BOSTON, MA 02115

DUDLEY, WILLIAM N, PHD
PROFESSOR
DEPARTMENT OF PUBLIC HEALTH EDUCATION
SCHOOL OF HEALTH AND
HUMAN PERFORMANCE
UNIVERSITY OF NORTH CAROLINA
GREENSBORO, NC 27402

ELDER, CHARLES R, MD *
AFFILIATE INVESTIGATOR
DEPARTMENT OF INTERNAL MEDICINE
AND CENTER FOR HEALTH RESEARCH
KAISER PERMANENTE NORTHWEST
PORTLAND, OR 97227

EPSTEIN, LEONARD H, PHD
PROFESSOR
DEPARTMENT OF PEDIATRICS
SOCIAL AND PREVENTIVE MEDICINE
STATE UNIVERSITY OF NEW YORK AT BUFFALO
BUFFALO, NY 14214

FISCELLA, KEVIN , MD *
PROFESSOR
DEPARTMENT OF FAMILY MEDICINE
SCHOOL OF MEDICINE AND DENTISTRY
UNIVERISTY OF ROCHESTER
ROCHESTER, NY 14620

LEVINE, MICHELE D, PHD
ASSOCIATE PROFESSOR
DEPARTMENT OF PSYCHIATRY
UNIVERSITY OF PITTSBURGH
PITTSBURGH, PA 15213

LI, FUZHONG , PHD
SENIOR RESEARCH SCIENTIST
OREGON RESEARCH INSTITUTE
EUGENE, OR 97403

MORLAND, KIMBERLY B, PHD
ASSOCIATE PROFESSOR
DEPARTMENT OF PREVENTIVE MEDICINE
MOUNT SINAI SCHOOL OF MEDICINE
NEW YORK, NY 10029

ODOMS-YOUNG, ANGELA M, PHD *
ASSISTANT PROFESSOR
DEPARTMENT OF KINESIOLOGY
AND NUTRITION
UNIVERSITY OF ILLINOIS AT CHICAGO
CHICAGO, IL 60612

OMAN, ROY F, PHD
PROFESSOR
DEPARTMENT OF HEALTH
PROMOTION SCIENCES
HEALTH SCIENCES CENTER
UNIVERSITY OF OKLAHOMA
OKLAHOMA CITY, OK 73190

POLLAK, KATHRYN I, PHD
ASSOCIATE PROFESSOR
DUKE CANCER PREVENTION
DETECTION AND CONTROL RESEARCH PROGRAM
DUKE UNIVERSITY MEDICAL CENTER
DURHAM, NC 27705

QUARELLS, RAKALE COLLINS, PHD
RESEARCH ASSOCIATE PROFESSOR
SOCIAL EPIDEMIOLOGY RESEARCH CENTER
MOREHOUSE SCHOOL OF MEDICINE
ATLANTA, GA 30310

RITENBAUGH, CHERYL KAY, PHD *
PROFESSOR
DEPARTMENT OF FAMILY
AND COMMUNITY MEDICINE
UNIVERSITY OF ARIZONA
TUCSON, AZ 85719

RITTERBAND, LEE M, PHD *
ASSOCIATE PROFESSOR
DEPARTMENT OF PSYCHIATRY AND
NEUROBEHAVIORAL SCIENCES
UNIVERSITY OF VIRGINIA HEALTH SYSTEM
CHARLOTTESVILLE, VA 22908

SHERWOOD, NANCY E, PHD
SENIOR RESEARCH INVESTIGATOR
HEALTH PARTNERS
INSTITUTE FOR EDUCATION AND
RESEARCH
BLOOMINGTON, MN 55425

SMITH, MICHAEL T, PHD
PROFESSOR
DEPARTMENT OF PSYCHIATRY, BEHAVIORAL SLEEP
MEDICINE PROGRAM, CENTER FOR BEHAVIOR
AND HEALTH, SCHOOL OF MEDICINE
BAYVIEW MEDICAL CENTER JOHNS HOPKINS
UNIVERSITY
BALTIMORE, MD 21224

STICE, ERIC M, PHD
SENIOR RESEARCH SCIENTIST
OREGON RESEARCH INSTITUTE
EUGENE, OR 97403

STUDTS, JAMIE L, PHD *
ASSOCIATE PROFESSOR
DEPARTMENT OF BEHAVIORAL
SCIENCE
COLLEGE OF MEDICINE
UNIVERSITY OF KENTUCKY
LEXINGTON, KY 40536

TATE, DEBORAH F, PHD
ASSOCIATE PROFESSOR
DEPARTMENT OF HEALTH BEHAVIOR
DEPARTMENT OF NUTRITION
UNIVERSITY OF NORTH CAROLINA
CHAPEL HILL, NC 27599

THOMPSON, DEBORAH I, PHD
ASSOCIATE PROFESSOR
USDA/ARS SCIENTIST/NUTRITIONIST
CHILDREN'S NUTRITION RESEARCH CENTER
BAYLOR COLLEGE OF MEDICINE
HOUSTON, TX 77030

WILFLEY, DENISE ELLA, PHD *
PROFESSOR
DEPARTMENT OF PSYCHIATRY,
PEDIATRICS, AND PSYCHOLOGY
SCHOOL OF MEDICINE IN ST. LOUIS
WASHINGTON UNIVERSITY
ST. LOUIS, MO 63110

YAROCH, AMY L, PHD
EXECUTIVE DIRECTOR
GRETCHEN SWANSON
CENTER FOR NUTRITION
OMAHA, NE 68105

MAIL REVIEWER(S)

MORRIS, CINDY ANNE, PHD
PROFESSOR
DEPARTMENT OF MICROBIOLOGY
AND IMMUNOLOGY
HEALTH SCIENCE CENTER
TULANE UNIVERSITY
NEW ORLEANS, LA 70112

SCIENTIFIC REVIEW ADMINISTRATOR

FITZSIMMONS, STACEY , PHD
SCIENTIFIC REVIEW OFFICER
CENTER FOR SCIENTIFIC REVIEW
NATIONAL INSTITUTES OF HEALTH
BETHESDA, MD 20892

GRANTS TECHNICAL ASSISTANT

JENNINGS, CHRISOULA , BS
LEAD EXTRAMURAL SUPPORT ASSISTANT
CENTER FOR SCIENTIFIC REVIEW
NATIONAL INSTITUTES OF HEALTH
BETHESDA, MD 20892

* Temporary Member. For grant applications, temporary members may participate in the entire meeting or may review only selected applications as needed.

Consultants are required to absent themselves from the room during the review of any application if their presence would constitute or appear to constitute a conflict of interest.
