# Supplementary material for: Effects of Bluetooth-Enabled Desk Ellipticals on Office Work Performance: Rationale, Design, and Protocol for a Randomized Trial With Overweight and Obese Adults
Source: JMIR Res Protoc. 2020 Jan 14;9(1):e16275. doi: 10.2196/16275 (PMC6996735; doi:10.2196/16275)
Supplement: Multimedia Appendix 2 [file resprot_v9i1e16275_app2.pdf]

SUMMARY STATEMENT
(Privileged Communication)

Release Date: 11/01/2013

PROGRAM CONTACT:
Charlotte Pratt
(301) 435-0382
prattc@nhlbi.nih.gov

Application Number: 1 R21 HL118453-01A1

Principal Investigator

ROVNIAK, LIZA PHD

Applicant Organization: PENNSYLVANIA STATE UNIVERSITY

Review Group: PRDP
Psychosocial Risk and Disease Prevention Study Section

Meeting Date: 09/16/2013
Council: JAN 2014
Requested Start: 04/01/2014

RFA/PA: PA12-179
PCC: HHCG N
Dual PCC: NLM DUAL
Dual IC(s): DK

Project Title: Desk-Compatible Elliptical Device: Feasibility Evaluation

SRG Action: Impact Score: 12 Percentile: 3 +
Next Steps: Visit http://grants.nih.gov/grants/next_steps.htm
Human Subjects: 30-Human subjects involved - Certified, no SRG concerns
Animal Subjects: 10-No live vertebrate animals involved for competing appl.
Gender: 1A-Both genders, scientifically acceptable
Minority: 1A-Minorities and non-minorities, scientifically acceptable
Children: 1A-Both Children and Adults, scientifically acceptable
Clinical Research - not NIH-defined Phase III Trial

Project Year	Direct Costs Requested	Estimated Total Cost
1	150,000	228,520
2	125,000	190,434
TOTAL	275,000	418,954

ADMINISTRATIVE BUDGET NOTE: The budget shown is the requested budget and has not been adjusted to reflect any recommendations made by reviewers. If an award is planned, the costs will be calculated by Institute grants management staff based on the recommendations outlined below in the COMMITTEE BUDGET RECOMMENDATIONS section.

1R21HL118453-01A1 Rovniak, Liza

RESUME AND SUMMARY OF DISCUSSION: This application seeks support to test the feasibility of a desk compatible elliptical device to promote exercise at the worksite. This well written resubmitted application offers very high impact to address the public health challenge of sedentary lifestyles and was highly responsive to the previous critique. Reviewers were unanimously enthusiastic about this work noting many strengths: the exceptional significance of reducing worksite sedentary behavior; the exceptional investigators and research environment; the exceptionally novel approach to increasing worksite exercise inherent in an elliptical device installed at a worker's desk; the ready and inexpensive dissemination this work could have a significant and positive impact on occupational health; and again noted the well written, compelling lab and field trial, cross over design, excellent recruitment plan and detailed assessment of work productivity. One minor weakness that could readily be addressed was noted: the qualitative interview missed an opportunity to assess acceptability that could delineate motivational factors. Overall this is an exceptional, ambitious and impressive application with very high potential to change sedentary behaviors at the worksite.

DESCRIPTION (provided by applicant): The proposed research will investigate the feasibility of accomplishing simultaneous caloric expenditure and productive office work using a height-adapted elliptical device designed to be pedaled at a standard desk. Strategies for increasing energy expenditure without requiring extra time investment are greatly needed, as most US adults report lack of time for physical activity, and spend over half of their waking hours in sedentary behaviors. These low levels of energy expenditure contribute to an average weight gain of 1 pound per year among US adults-which raises risk for cardiovascular disease, metabolic syndrome, diabetes, and cancer. Pedaling a low-cost elliptical device while simultaneously completing office work could help reverse this trend toward weight gain-without requiring extra time investment to increase physical activity. However, there is a lack of research on the feasibility of accomplishing productive office work while using elliptical/pedaling devices. Evaluating the feasibility of simultaneous pedaling and productive office work is important for determining whether elliptical/pedaling devices should be more widely disseminated across diverse sedentary office settings. Therefore, the primary specific aims of this research are to: (1) assess the feasibility of completing simulated office work activities in a lab-based setting while pedaling the elliptical device at different intensity levels among sedentary adults varying on age, gender, and body mass index (Study 1, n = 112); and (2) assess the feasibility of completing sedentary desk work in a field-based (office) setting while pedaling the elliptical device at a self-selected intensity level, as well as social and built environment influences on elliptical device use (Study 2, n = 50). The proposed research builds upon established ecological models demonstrating the importance of proximal environmental influences on physical activity and sedentary behavior. The combination of lab- and field-based research will contribute to the internal and external validity of study findings, and help guide recommendations for integrating elliptical/pedaling devices in sedentary office settings. Even if used only part of the working day, elliptical devices could substantially increase daily caloric expenditure and contribute to reduced risk of obesity and chronic health conditions associated with sedentary lifestyles.

PUBLIC HEALTH RELEVANCE: The proposed research will explore the feasibility of accomplishing simultaneous energy expenditure and productive office work while pedaling a desk-compatible elliptical device. Unlike other existing solutions for promoting simultaneous office work and energy expenditure, the desk-compatible elliptical device is low cost, space-efficient, and can be used while seated at a standard-height work desk. Widespread use of low-cost desk-compatible elliptical devices, or other similar pedaling devices, could help prevent further growth of the obesity epidemic and reduce risk of cardiovascular disease, metabolic syndrome, diabetes, and some cancers.

CRITIQUE 1:

Significance: 2

Investigator(s): 2
Innovation: 2
Approach: 2
Environment: 2

Overall Impact: This revised application is designed to assess the feasibility of completing simulated desk work activities while using a desk-compatible elliptical exercise machine in both lab and field settings. Overall, the investigators were quite responsive to the concerns raised in the initial critique. The proposed project has several notable strengths including a significant objective, novel aims, strong pilot data, and well-articulated methodology. However, some concerns that detract from the potential impact of the project also remain. One concern is that in addressing key critique issues the investigators may have made the project too ambitious for the project resources and time period. Another concern is a missed opportunity to evaluate some additional assessments that could provide important information regarding the acceptability and feasibility of the elliptical. Overall, there is considerable merit to the proposed project which addresses an important issue in promoting physical activity with a novel focus and approach.

1. Significance:

Strengths

- The investigators were quite responsive to the issues raised in the initial critique. This strengthened the application and its potential impact considerably.
- If the aims are met, there is potential for meaningful public health impact through decreasing sedentary time at work during time typically spent inactive.
- The opportunity to overcome common PA barriers by bring a reasonable sophisticated, yet simple, form of PA into workplace is both significant and novel.
- Strong pilot data and a well-qualified investigative team heighten the likelihood of project success.

Weaknesses

- A modest concern is that in addressing key critique issues the investigators may have made the project too ambitious for the project resources and time period.
- The battery of feasibility measures lacks many subjective and/or perceptual measures that may help delineate keep motivational factors associated with acceptability, use, and adherence. This is a missed opportunity that can be easily addressed via inclusion of select psychosocial measures of affect, effort sense, cognition, & preference.

2. Investigator(s):

Strengths

- A strong, well-qualified investigative team with considerable prior experience has been assembled.

Weaknesses

- None noted.

3. Innovation:

Strengths

- The desk compatible elliptical is quite novel and could yield meaningful impact given some apparent strengths over other similar types of work-based exercise stations.
- The assessment of work productivity is also quite innovative.

Weaknesses

- None noted.

4. Approach:

Strengths

- Well-qualified, experienced investigative team has been assembled.
- Design is well articulated and appropriate to achieve the proposed study objectives.
- Revised definition of sedentary strengthens the application.
- Well justified measures for many key outcomes

Weaknesses

- A modest concern is that in addressing key critique issues the investigators may have made the project too ambitious for the project resources and timeframe. Notably the 112 participants proposed for the lab assessment and 1 month field assessment, while directly responsive to concerns raised initially, may be too ambitious.
- The battery of feasibility measures lacks many subjective and/or perceptual measures that may help delineate keep motivational factors associated with acceptability, use, and adherence. This is a missed opportunity that can be easily addressed via inclusion of select psychosocial measures of affect, effort sense, cognition, & preference. For example, inclusion of measures such as the feeling scale, enjoyment of using the elliptical, RPE scale, task self-efficacy would all be quite informative. Additionally, including cognitive and preference assessments tapping participants receptivity to use, plans for future use, perceived barriers to use, etc could all be very important in determining feasibility and preliminary efficacy.

5. Environment:

Strengths

- The environment is very strong and provides appropriate support and resources to ensure success of the project.

Weaknesses

- None noted.

Protections for Human Subjects:

- The protection procedures are sufficiently detailed and appropriate.

Data and Safety Monitoring Plan (Applicable for Clinical Trials Only):

Acceptable

- The DSMP is appropriate for the proposed investigation.

Inclusion of Women, Minorities and Children:

G1A - Both Genders, Acceptable

M1A - Minority and Non-minority, Acceptable

C1A - Children and Adults, Acceptable

Vertebrate Animals:

Not Applicable (No Vertebrate Animals)

Budget and Period of Support:

Recommend as requested

CRITIQUE 2:

Significance: 1
Investigator(s): 1
Innovation: 1
Approach: 1
Environment: 1

Overall Impact: The investigators aim to test the feasibility of accomplishing simultaneously caloric expenditure and productive office work using a height adapted elliptical device designed to be pedaled at a standard desk. The investigators aim to assess the feasibility in a lab based setting while pedaling at different intensity levels among a study population of sedentary adults with variation in age, gender, and BMI. In addition, the investigators have planned for a second study where the feasibility of completing desk work while pedaling the elliptical at self-selected intensity levels will be measured in office environments. The application is well written and lead by a strong investigative team. The studies are well designed and supported by previous research. If successful, the public health significance of this easy to use and economical intervention will be high in increasing physical activity within sedentary office environments.

1. Significance:

Strengths

- The investigators are addressing the current trend where work related caloric expenditure has decreased over the past several decades and many people in the work force are in sedentary office jobs.
- The aim to develop desk compatible elliptical devices makes increasing daily exercise easy and affordable.
- The desk compatible elliptical device is consistent with recommendations from both the CDC and American Heart Association.

Weaknesses

- None noted.

2. Investigator(s):

Strengths

- The study is led by a strong investigative team.

Weaknesses-

- None noted.

3. Innovation:

Strengths

- The device is innovative in that it is an improvement on existing workplace exercise devices in that it: (a) has the potential to be used without leaving one’s desk, increasing the convenience of use; and (b) is inexpensive and therefore feasible for employers to invest in if effective.
- It is innovative to measure the effect of the device use on work productivity as that would be a concern if the device were to be used with any regularity.

Weaknesses

- None noted.

4. Approach:

Strengths

- The design to conduct both a lab study where intensity levels and work performance measures can be controlled in addition to an observational study where the effect of self- selected intensity on work performance is a strength.
- The work performance measures seem acceptable.
- The measurement of future interest in the use of the elliptical is a strength in measuring the feasibility of this type of device use in work environments.
- The use of an Actigraph for measurement of the elliptical use is a strength in the second aim.
- The measurement of the social influences within real work environments is also a strength.
- The analytical plans for both aims are well described.

Weaknesses –

- None noted.

5. Environment:

Strengths

- The environment at Pennsylvania State University is well suited for the research.

Weaknesses

- None noted.

Protections for Human Subjects:

Acceptable Risks and/or Adequate Protections

Data and Safety Monitoring Plan (Applicable for Clinical Trials Only):

Acceptable. No concerns.

Inclusion of Women, Minorities and Children:

G1A - Both Genders, Acceptable

M1A - Minority and Non-minority, Acceptable

C1A – Children and Adults Included, Acceptable

Vertebrate Animals:

Not Applicable (No Vertebrate Animals)

Biohazards:

Not Applicable (No Biohazards)

Resubmission:

- The investigators were very responsive to the previous review.

Budget and Period of Support:

Recommend as Requested

CRITIQUE 3:

Significance: 1
Investigator(s): 1
Innovation: 1
Approach: 2
Environment: 1

Overall Impact: This is a very well written and highly responsive resubmission. The proposal is both quite significant and innovative and could lead to the development of an inexpensive disseminable weight prevention intervention that could be readily incorporated into many workplace environments. The proposal is highly significant in that it targets workday sedentarism. Fifty percent of workers sit behind computers for long periods of time during the day, which has been estimated to have reduced average caloric daily expenditure by 100 calories/day since 1960. The project tests the feasibility of an elliptical device that has been ergonomically modified by the team to be used while sitting at a desk doing office work. This overcomes the most common barrier to PA, which is competing time demands. The device would have major advantages over desk treadmill devices with respect to cost ($100), space, and user physical ability requirements. The project is well conceived and has many strengths and negligible weaknesses. With respect to significance, it is estimated that if the device were used for 2 hours per day, 5 days/week for 48 weeks, it would be possible to lose 7 pounds per year, holding all else constant. If the project is successful in demonstrating that the device does not interfere with workplace productivity, the potential for uptake would be very high. It would support large scale clinical trials to test health outcomes, which could have tremendous impact on the obesity epidemic at the population level. In addition to significance of the concept, the project design is outstanding, including both a highly controlled lab experiment and a field study. The lab based study uses the superb Latin square design to test whether 4 different pedaling intensities impact tests of typing speed and cognitive performance. The team has strong and innovative pilot data having adapted a low cost elliptical device for use under a standard desk, invented a portable power output measurement system and tested caloric expenditure in 32 sedentary adults. The proposed field study has been extended to one month and includes a qualitative interview of user impressions, which should provide important usability data

for refinement in larger trials. The team and environment are excellent. A very minor weakness is lack of direct feedback from colleagues and supervisors related to enthusiasm, workplace disruption, etc.

Protections for Human Subjects:

Acceptable Risks and/or Adequate Protections

- Risk are minimal in studying low intensity PA in a healthy sample

Data and Safety Monitoring Plan (Applicable for Clinical Trials Only):

Acceptable

- There is plan for an internal DSMB. Formal criteria for coding adverse event severity and defining anticipated AES should be added

Inclusion of Women, Minorities and Children:

G1A - Both Genders, Acceptable

M1A - Minority and Non-minority, Acceptable

C1A - Children and Adults, Acceptable

- A minimum 25 percent minority inclusion will be sought. 20 year old children will be included. Not clear why 18 and 19 year olds will be excluded

Vertebrate Animals:

Not Applicable (No Vertebrate Animals)

Biohazards:

Not Applicable (No Biohazards)

Resubmission:

- This is a resubmitted application. The investigators have operationalized their definition of feasibility. They have modified their eligibility criteria to include a more conservative definition of sedentary (<60 mins. of PA/week and engaging in sedentary office work at least 6 hours/day). They have extended the length of the field trial from 5 days to 1 month and added a qualitative assessment. They have also added considerable qualitative expertise to the team (Drs. Morse & Rothrock). This is a highly responsive resubmission.

Budget and Period of Support:

Recommend as Requested

THE FOLLOWING RESUME SECTIONS WERE PREPARED BY THE SCIENTIFIC REVIEW OFFICER TO SUMMARIZE THE OUTCOME OF DISCUSSIONS OF THE REVIEW COMMITTEE ON THE FOLLOWING ISSUES:

PROTECTION OF HUMAN SUBJECTS (Resume): ACCEPTABLE. There are no concerns.

INCLUSION OF WOMEN PLAN (Resume): ACCEPTABLE. Both females and males will be recruited equally for this study.

INCLUSION OF MINORITIES PLAN (Resume): ACCEPTABLE. The target enrollment estimates inclusion of all race and ethnic groups.

INCLUSION OF CHILDREN PLAN (Resume): ACCEPTABLE. Children aged 20 and adults will be recruited for this study.

COMMITTEE BUDGET RECOMMENDATIONS: The budget was recommended as requested.

+ Derived from the range of percentile values calculated for the study section that reviewed this application.

NIH has modified its policy regarding the receipt of resubmissions (amended applications). See Guide Notice NOT-OD-10-080 at http://grants.nih.gov/grants/guide/notice-files/NOT-OD-10-080.html.
The impact/priority score is calculated after discussion of an application by averaging the overall scores (1-9) given by all voting reviewers on the committee and multiplying by 10. The criterion scores are submitted prior to the meeting by the individual reviewers assigned to an application, and are not discussed specifically at the review meeting or calculated into the overall impact score. Some applications also receive a percentile ranking. For details on the review process, see http://grants.nih.gov/grants/peer_review_process.htm#scoring.

MEETING ROSTER

Psychosocial Risk and Disease Prevention Study Section
Risk, Prevention and Health Behavior Integrated Review Group
CENTER FOR SCIENTIFIC REVIEW
PRDP
September 16, 2013 - September 17, 2013

CHAIRPERSON

EPSTEIN, LEONARD H, PHD
SUNY DISTINGUISHED PROFESSOR
DEPARTMENT OF PEDIATRICS
DIVISION OF BEHAVIORAL MEDICINE
STATE UNIVERSITY OF NEW YORK AT BUFFALO
BUFFALO, NY 14214

MEMBERS

ABRANTES, ANA M, PHD
PROFESSOR
DEPARTMENT OF PSYCHIATRY AND
HUMAN BEHAVIOR, ALPERT MEDICAL
SCHOOL, BUTLER HOSPITAL
BROWN UNIVERSITY
PROVIDENCE, RI 02906

AYALA, GUADALUPE X, PHD
PROFESSOR
INSTITUTE FOR BEHAVIORAL AND
COMMUNITY HEALTH
SAN DIEGO STATE UNIVERSITY
SAN DIEGO, CA 92123

BAEZCONDE-GARBANATI, LOURDES ALBERTINA, MPH, PHD
ASSOCIATE PROFESSOR
DEPARTMENT OF PREVENTIVE MEDICINE
AND SOCIOLOGY, KECK SCHOOL OF MEDICINE
NORRIS COMPREHENSIVE CANCER CENTER
UNIVERSITY OF SOUTHERN CALIFORNIA
LOS ANGELES, CA 90033

BASKIN, MONICA L, PHD
ASSOCIATE PROFESSOR
DIVISION OF PREVENTIVE MEDICINE
UNIVERSITY OF ALABAMA AT BIRMINGHAM
BIRMINGHAM, AL 35294

BOUTELLE, KERRI N, PHD
PROFESSOR
DEPARTMENT OF PEDIATRICS
AND PSYCHIATRY
UNIVERSITY OF CALIFORNIA, SAN DIEGO
LA JOLLA, CA 92037

COUPS, ELLIOT J, PHD
ASSOCIATE PROFESSOR
DEPARTMENT OF HEALTH EDUCATION
RUTGERS, THE STATE UNIVERSITY OF NEW JERSEY
ROBERT WOOD JOHNSON MEDICAL SCHOOL
NEW BRUNSWICK, NJ 08901

DAVISON, KIRSTEN , PHD
ASSOCIATE PROFESSOR
DEPARTMENT OF NUTRITION
HARVARD SCHOOL OF PUBLIC HEALTH
BOSTON, MA 02115

DODD, VIRGINIA JONES, PHD *
ASSOCIATE PROFESSOR
DEPARTMENT OF COMMUNITY DENTISTRY
AND BEHAVIORAL SCIENCES
UNIVERSITY OF FLORIDA
GAINESVILLE, FL 32611

FERRY, ROBERT , MD *
PROFESSOR AND CHIEF
DIVISION OF PEDIATRIC
DEPARTMENT OF ENDOCRINOLOGY AND METABOLISM
LE BONHEUR CHILDRENS HOSPITAL
UNIVERSITY OF TENNESSEE HEALTH SCIENCES
CENTER
MEMPHIS, TN 38103

FOCHT, BRIAN CARL, PHD *
ASSOCIATE PROFESSOR
DEPARTMENT OF KINESIDOLOGY
COMPREHENSIVE CANCER CENTER
AND SOLOVE RESEARCH INSTITUTE
THE OHIO STATE UNIVERSITY
COLUMBUS, OH 43210

LEAHEY, TRICIA M, PHD
ASSISTANT PROFESSOR
WEIGHT CONTROL AND DIABETES RESEARCH CENTER
MIRIAM HOSPITAL
BROWN MEDICAL SCHOOL
PROVIDENCE, RI 02903

LEVINE, MICHELE D, PHD
ASSOCIATE PROFESSOR
DEPARTMENT OF PSYCHIATRY
UNIVERSITY OF PITTSBURGH
PITTSBURGH, PA 15213

MADSEN, KRISTINE A, MD *
ASSISTANT PROFESSOR
SCHOOL OF PUBLIC HEALTH, JOINT MEDICAL PROGRAM
AND PUBLIC HEALTH NUTRITION
UNIVERSITY OF CALIFORNIA BERKELEY
BERKELEY , CA 94720

MORLAND, KIMBERLY B, PHD
ASSOCIATE PROFESSOR
DEPARTMENT OF PREVENTIVE MEDICINE
MOUNT SINAI SCHOOL OF MEDICINE
NEW YORK, NY 10029

PAGOTO, SHERRY L, PHD *
ASSISTANT PROFESSOR
DIVISION OF PREVENTIVE AND BEHAVIORAL MEDICINE
DEPARTMENT OF MEDICINE
UNIVERSITY OF MASSACHUSETTS MEDICAL SCHOOL
WORCESTER, MA 01655

PAUL, IAN M, MD *
PROFESSOR
DEPARTMENT OF PEDIATRICS
PENNSYLVANIA STATE COLLEGE OF MEDICINE
HERSHEY, PA 17033-085

POLLAK, KATHRYN I, PHD
ASSOCIATE PROFESSOR
DUKE CANCER PREVENTION
DETECTION AND CONTROL RESEARCH PROGRAM
DUKE UNIVERSITY MEDICAL CENTER
DURHAM, NC 27705

QUARELLS, RAKALE COLLINS, PHD
RESEARCH ASSOCIATE PROFESSOR
SOCIAL EPIDEMIOLOGY RESEARCH CENTER
MOREHOUSE SCHOOL OF MEDICINE
ATLANTA, GA 30310

SMITH, MICHAEL T, PHD
PROFESSOR
DEPARTMENT OF PSYCHIATRY AND BEHAVIORAL SLEEP
CENTER FOR BEHAVIOR AND HEALTH
SCHOOL OF MEDICINE
JOHNS HOPKINS UNIVERSITY
BALTIMORE, MD 21224

SPRING, BONNIE , PHD *
PROFESSOR
DEPARTMENT OF PREVENTIVE MEDICINE
NORTHWESTERN UNIVERSITY
CHICAGO, IL 60611

TATE, DEBORAH F, PHD
ASSOCIATE PROFESSOR
DEPARTMENT OF HEALTH BEHAVIOR
AND NUTRITION
UNIVERSITY OF NORTH CAROLINA
CHAPEL HILL, NC 27599

THOMPSON, DEBORAH I, PHD
ASSOCIATE PROFESSOR
USDA/ARS SCIENTIST/NUTRITIONIST
CHILDREN'S NUTRITION RESEARCH CENTER
BAYLOR COLLEGE OF MEDICINE
HOUSTON, TX 77030

WILFLEY, DENISE ELLA, PHD
PROFESSOR
DEPARTMENT OF PSYCHIATRY, MEDICINE
PEDIATRICS, AND PSYCHOLOGY
SCHOOL OF MEDICINE IN ST. LOUIS
WASHINGTON UNIVERSITY
ST. LOUIS, MO 63110

YAROCH, AMY L, PHD
EXECUTIVE DIRECTOR
GRETCHEN SWANSON
CENTER FOR NUTRITION
OMAHA, NE 68105

MAIL REVIEWER(S)

STICE, ERIC M, PHD
SENIOR RESEARCH SCIENTIST
OREGON RESEARCH INSTITUTE
EUGENE, OR 97403

SCIENTIFIC REVIEW ADMINISTRATOR

FITZSIMMONS, STACEY , PHD
SCIENTIFIC REVIEW OFFICER
CENTER FOR SCIENTIFIC REVIEW
NATIONAL INSTITUTES OF HEALTH
BETHESDA, MD 20892

GRANTS TECHNICAL ASSISTANT

NJOKU, PHILIP C
CENTER FOR SCIENTIFIC REVIEW
NATIONAL INSTITUTES OF HEALTH
BETHESDA, MD 20892

* Temporary Member. For grant applications, temporary members may participate in the entire meeting or may review only selected applications as needed.

Consultants are required to absent themselves from the room during the review of any application if their presence would constitute or appear to constitute a conflict of interest.
